# Construction and validation of an educational booklet for patients in the postoperative period of cardiac surgery: a methodological study

**DOI:** 10.1590/0034-7167-2022-0621

**Published:** 2023-12-04

**Authors:** Sônia Regina Barcellos, Alexandre do Rosário Joras, Angelita Paganin Constanzi, Emiliane Nogueira de Souza

**Affiliations:** I Universidade de Caxias do Sul. Caxias do Sul, Rio Grande do Sul, Brazil; II Hospital Pompéia. Caxias do Sul, Rio Grande do Sul, Brazil; III Universidade Federal de Ciências da Saúde de Porto Alegre. Porto Alegre, Rio Grande do Sul, Brazil; IV Hospital da Unimed Nordeste. Caxias do Sul, Rio Grande do Sul, Brazil

**Keywords:** Health Education, Cardiac Surgical Procedures, Nursing Care, Self-Care, Cardiology, Educación para la Salud, Procedimientos Quirúrgicos Cardíacos, Cuidados de Enfermería, Autocuidado, Cardiología, Educação em Saúde, Procedimentos Cirúrgicos Cardíacos, Cuidados de Enfermagem, Autocuidado, Cardiologia

## Abstract

**Objective:**

to construct and validate an educational booklet for self-care of patients in the postoperative period of cardiac surgery

**Methods:**

methodological study, including bibliographic survey, construction of the booklet and validation with judges and the target audience. For validation with judges, the Health Educational Content Validation Instrument was used, and with the target audience, an instrument was used with questions related to organization, writing style, appearance and motivation. To analyze the judges’ answers, the content validation index was used

**Results:**

the booklet was prepared with 14 topics. The content validation index among the eight judges was 1 and the concordance index among the ten patients was above 80%. The final version of the material was made available in printed format

**Conclusion:**

the educational booklet was developed and validated by judges and the target audience, serving as an educational support tool for self-care of patients in the postoperative period of cardiac surgery.

## RESUMO

**Objetivo:**

construir e validar uma cartilha educativa para o autocuidado de pacientes no pós-operatório de cirurgia cardíaca.

**Métodos:**

estudo metodológico, incluindo levantamento bibliográfico, construção da cartilha e validação com juízes e com o público-alvo. Para validação com juízes, utilizou-se o Instrumento de Validação de Conteúdo Educativo em saúde e com o público-alvo utilizou-se um instrumento com questões relacionadas à organização, estilo da escrita, aparência e motivação. Para análise das respostas dos juízes foi utilizado o índice de validação de conteúdo.

**Resultados:**

a cartilha foi elaborada com 14 tópicos. O índice de validação de conteúdo entre os oito juízes foi de 1 e o índice de concordância entre os dez pacientes acima de 80%. A versão final do material foi disponibilizada no formato impresso.

**Conclusão:**

a cartilha educativa foi desenvolvida e validada por juízes e público-alvo, servindo como uma ferramenta educativa de apoio para o autocuidado de pacientes no pós-operatório de cirurgia cardíaca.

## RESUMEN

**Objetivo:**

construir y validar un folleto educativo para el autocuidado de pacientes en postoperatorio de cirugía cardíaca.

**Métodos:**

estudio metodológico, con levantamiento bibliográfico, construcción del folleto y validación por jueces y público-objeto. Para la validación de los jueces, se utilizó el Instrumento de Validación del Contenido Educativo en salud y con el público-objeto, un instrumento con preguntas relacionadas a la organización, estilo de la escritura, apariencia y motivación. Para analizar las respuestas de los jueces, se utilizó el índice de validación del contenido.

**Resultados:**

el folleto se elaboró con 14 temas. El índice de validación entre los ocho jueces fue 1 y el índice de acuerdo entre los diez pacientes, superior al 80%. La versión final se puso a disposición en formato impreso.

**Conclusión:**

el folleto educativo fue desarrollado y validado por jueces y público objeto como herramienta educativa para apoyar el autocuidado de pacientes en el postoperatorio de cirugía cardíaca.

## INTRODUCTION

Cardiovascular diseases stand out worldwide due to the high mortality rates^(1)^. Among the cardiac diseases, ischemic heart disease is highlighted, which is the main cause of death in the world and also in Brazil^(2)^, whose treatment possibilities include medication, percutaneous coronary intervention and surgery. Among cardiac surgeries, myocardial revascularization (CABG) has the highest prevalence (64% in Brazil) with the aim of restoring blood flow in the coronary arteries^(3)^. Valvular surgery, whether for reconstructive or replacement purposes, corresponds to approximately 20% of cardiac surgeries, and is indicated when there are valve diseases that compromise cardiac function^(4-6)^.

In recent years, a change in the clinical profile of patients has been noticed. Due to the increase in life expectancy, surgical procedures have been indicated for individuals of more advanced age groups. Thus, surgical indication in older adults who already have multiple associated comorbidities influences clinical outcomes and postoperative complications^(7-8)^. Male older adults, whose most prevalent comorbidities are arterial hypertension (AH), diabetes mellitus (DM) and dyslipidemia, mostly characterize patients undergoing cardiac surgery^(4,6)^. For these patients, surgical treatment is an option, the objective of which is to increase survival rate and improve quality of life^(9)^. However, cardiac surgery is considered a highly complex procedure, which causes a socioeconomic and cultural impact on the lives of patients, generating a demand for adaptation to a new health condition, which results in coping with the need for changes in lifestyle^(10)^.

In this context, self-care becomes a priority for patients and, despite the importance of the support network, it is necessary to encourage self-care, autonomy and independence^(10)^. Physical and emotional changes are also frequently reported by patients, and can become obstacles in patients’ daily lives, making it difficult, for example, to regularly practice physical exercises, which is recommended for cardiovascular rehabilitation^(11)^. So, it is in the hospital context, in the postoperative period during hospitalization, that it is necessary to start preparing for discharge with a view to a satisfactory recovery, reduction of complications, incidence of infection and readmission, as well as the resumption of daily life and self-care. Discharge planning aims to reduce perceived barriers and difficulties in the disease and hospitalization process, as well as to promote self-care activities. However, frequently, patients are discharged from the hospital and return home anxious, insecure and with doubts, thus highlighting the need to strengthen the preparation of the family-patient unit for self-care at home^(12)^. The focus of the nurse’s educational process should be on those doubts that occur after cardiac surgery, making it necessary to direct efforts to understand the patient’s learning needs to plan the discharge in an individualized way^(13-14)^. Higher levels of knowledge about health problems that encourage physical and emotional self-care actions have been associated with the use of educational resources^(12)^.

Greater safety is promoted for the patient and family, providing information and clarification of doubts, so that the care team can ensure the continuity of care at home. And the use of support resources, such as printed materials, can contribute to better health literacy and, consequently, decision-making aimed at better results. However, it is necessary to consider, in addition to the quality of the content, the language used, the layout of the text, illustrations and visual communication so that it is attractive and facilitates consultation and learning. For this purpose, the validation of the material by experienced professionals in the subject and by the target audience itself is used. It is known that not all institutions that have highly complex cardiology services develop adequate preparation for hospital discharge or provide educational resources for their patients. Thus, this study was developed to build an educational resource to support the verbal guidance provided by the health team to patients undergoing highly complex procedures in a hospital environment, with a view to promoting self-care after hospital discharge.

## OBJECTIVE

Build and validate an educational booklet for self-care of patients in the postoperative period of cardiac surgery.

## METHODS

### Ethical aspects

The study was approved by the Research Ethics Committee, ensuring compliance with the recommendations of Resolution 466/12, receiving a favorable opinion. All participants (judges and patients) received and signed the Informed Consent Form (ICF).

### Study design, period and setting

This is methodological research developed in three stages: construction of the educational booklet, content validation with judges and validation with the target audience, carried out from October 2020 to April 2021, by two nurses from the cardiac surgery service from a large general hospital in southern Brazil, a professor and a nursing undergraduate student. This study follows the guidelines of the Revised Standards for Quality Improvement Reporting Excellence (SQUIRE 2.0). The SQUIRE guidelines provide a framework for reporting new knowledge about how to improve health care.

### Construction of the booklet

A narrative review of the literature was carried out in which the selection of information took place in line with the researchers’ theoretical-practical experience on the subject. The review was carried out in the databases and virtual libraries: Latin American and Caribbean Literature in Health Sciences (LILACS), PubMed®, Scientific Electronic Library Online (Scielo). Research papers were selected according to the guiding question: What information is needed for the self-care of patients undergoing cardiac surgery (myocardial or valve revascularization) after hospital discharge? The following descriptors were used for research: Health education; Cardiac surgical procedures; Self-care. Inclusion criteria were: publication from 2015, in Portuguese and English. Exclusion criteria: theses, dissertations and monographs. A total of 5 articles were included. Guidelines^(15-16)^ of the Brazilian Society of Cardiology were also consulted, with the aim of identifying necessary care in the postoperative period of myocardial revascularization and valve surgery. The publications underwent critical reading in order to extract useful information to be included in the booklet, defining the main topics and their objectives.

From then on, the texts of each topic were prepared, with a reader-centered approach, bringing clear and objective information, based on resources aimed at health literacy from the Centers for Medicare and Medicaid Service^(17)^, which presents guidelines aimed at language, illustration and graphic layout of the material, as well as recommendations regarding illustrative designs, layout, formatting and configurations. The presentation of the booklet for validation was in accordance with the color palette that makes up elements of the institution’s visual identity. The booklet was submitted to content and appearance evaluation by experts (judges) in the subject.

### Sample and eligibility criteria for validation

For the selection of content judges, it was stipulated that they should meet at least two requirements described by Jasper^(18)^, so that they could be considered as specialists in the subject: having professional experience assisting with patients in the postoperative period of cardiac surgery for a minimum period of 2 years; Having specialized skills/knowledge (training) that make the professional a reference in the subject. To list potential judges, consultations were carried out on the Lattes Platform of the National Council for Scientific and Technological Development (CNPq) and judges (health professionals) from different areas of activity were selected through intentional non-probabilistic sampling. After identifying the judges, an invitation was sent by e-mail to eleven professionals to participate in the study with guidance on the validation process.

For the validation of the booklet by the target audience, during the study period, patients in the postoperative period of cardiac surgery, admitted to the institution’s hospitalization unit, who were in pre-hospital discharge (between the 5th and 7th day after surgery), according to the following criteria: being clinically stable, being lucid and oriented in time and space, and showing willingness to read the booklet. Patients with some inability to read or evaluate the material were excluded. The selection of surgical patients (post-operative CABG or valve surgery) took place consecutively, as the procedures took place at the institution and the patients were already able to be discharged, in the referred period of study development.

### Validation of judges

For the validation of the judges, the Health Education Content Validation Instrument (HECVI) was used^(19)^, which is composed of two parts: the first contains the judges’ identification data and their professional experience. The second part contains the instructions for completing the instrument and the evaluative items of the booklet, namely: five questions addressing the objectives, ten questions about structure and presentation, and three questions related to relevance. The answers to each question are Likert-type with scores from one to four: (1) not relevant, (2) slightly relevant, (3) very relevant, and (4) extremely relevant. The ICF and the instrument were sent by e-mail to the judges who agreed to participate in the study, with guidance on the evaluation process.

### Validation of the target audience

Validation with the target public also took place during hospitalization, when a nurse approached the patient in the postoperative period by inviting them. When accepted, each patient or family member was given an envelope containing the booklet and a semi-structured questionnaire with questions related to sociodemographic data and the instrument for validation. The instrument could be completed by the patient himself or with the help of a family member and later collected by the ward nurse. The instrument used to validate the target audience, adapted from a previous study^(20)^, contains 13 questions that were grouped according to organization, writing style, appearance and motivation. The answer options for each question were categorized as follows: positive answers (yes/clear/interesting/understandable), negative answers (no/confused/don’t know/uninteresting/complicated).

### Results and statistical analysis

For the analysis of the responses of the HECVI instrument, the content validation index (CVI) was used, which measures the proportion of judges in agreement. The agreement between judges occurs when two or more evaluators evaluate the results in an equivalent way^(21)^. The calculation of the CVI of each item of the Health Education Content Validation instrument (HECVI)^(19)^ is performed by adding the items that were marked with “3” or “4” by the judges, dividing the result of this sum by the total number of responses obtained for the item. Thus, the items that received a “1” or “2” score should be revised or deleted. Items that received a score of “3” or “4” were considered as a positive result. In this study, a minimum acceptable CVI of 0.80 was considered^(21)^.

To analyze the responses of the instrument used by the patients, the agreement index (AI)^(20)^ was used, which consists of calculating the number of times the patients agreed, dividing it by the total number of evaluations (varies between 0 and 1). For this study, agreement in positive responses greater than or equal to 0.8 was considered satisfactory. The analysis of sociodemographic data was performed using descriptive statistics, presented in n(%) or mean and standard deviation.

## RESULTS

### Construction of the booklet

A Word® file with all the content was sent to the institution’s Marketing sector, which carried out the layout, based on the authors’ requests. The booklet was prepared in A5 paper size (148x210mm) in a pre-validation version with 20 pages, colored according to the visual identity of the hospital institution. Once this version was approved by the authors, 10 full-color copies were sent for printing, to then be made available for validation by the judges and, subsequently, with the patients. The content of the booklet covers all the topics presented in Chart 1, using figures available on the internet, all copyright free (free image bank). After validation by the judges, changes were made to the material and then 10 more copies were printed and then validated with the target audience.

**Chart 1:** Topics that make up the booklet and its objectives

Topics of the booklet	Objective
Myocardial revascularization surgery	To present the main objective of this procedure and to illustrate with figures
Valve surgery	To present the types of valve surgeries, as well as the types of prostheses, pros and cons of each material and alert for surgical or dental procedures; Point out the need for attention for signs of infection.
Preparations for returning home	To highlight the minimum requirements to be discharged from hospital, the general care needed to be taken at home soon after hospital discharge and medication intake; To address the occurrence of discomfort as the person integrates into everyday activities.
Operative wound	To addressing the healing process of the surgical wound and necessary care during bathing, dressing and sun exposure; To highlight the importance of detecting signs of infection.
Postoperative rehabilitation	To present the main actions that contribute to adequate postoperative rehabilitation, including professional follow-up when necessary.
Physical activity	To highlight the benefits of performing physical activity in rehabilitation and secondary prevention in the case of ischemic heart disease; To exemplify the recommended exercises according to the individual’s profile.
Day to day activities	To guide the performance of day-to-day activities according to the postoperative period, guiding family members and patients regarding the type of activity and intensity.
Sleep	To address symptoms that may occur, and preventive care to avoid or alleviate them.
Sex life	To approach the gradual return of sexual activity so that men can have part of their doubts clarified.
Appetite	To highlight the need to maintain a healthy diet.
Diabetes	To advise on the need for glycemic control to contribute to a better recovery, thus avoiding possible complications.
Drug consumption	To address smoking and alcoholism as factors that impair health and postoperative recovery.
Anticoagulation	To inform patients about precautions with the use of anticoagulant drugs and the risks.
Support network	To highlight the importance of keeping in touch with family and friends, avoiding being alone in the first few weeks.

### Validation of the booklet

The booklet was validated by eight expert judges who agreed to participate in the study. The judges were predominantly female 5 (62.5%), with a mean age of 39.12±6.9 years, four physicians, two nurses, one physiotherapist and one nutritionist. The average time of professional experience with cardiac surgery patients of professionals was 11.25±8.10 years.

In the validation process of the booklet, the items related to the objectives of the educational material had 35 (87.5%) responses marked as very relevant and 5 (12.5%) very relevant, maintaining a CVI of 1.0. Data presented in Table 1.

**Table 1:** Evaluation of the content judges regarding the objectives of the educational material (N=08). Porto Alegre, Rio Grande do Sul, Brazil, 2021

Objective	Extremely important	Very important	CVI
Consider the proposed theme	0	8	1.0
Suitable for the teaching-learning process	0	8	1.0
Clarifies doubts about the topic addressed	1	7	1.0
Provides reflection on the topic	1	7	1.0
Encourages behavior change	3	5	1.0

*CVI= Content Validity Index.*

The judges evaluated the booklet regarding the structure and presentation of the material, with regard to the language, clarity and sequence of information and the text. Thus, 65(81.25%) of the answers were marked as extremely relevant and 15(18.75%) as very relevant. No item was considered irrelevant or slightly relevant, the booklet was considered validated, maintaining a CVI of 1.0 as shown in Table 2.

**Table 2:** Evaluation of the judges regarding the structure and presentation of the educational material (N=08). Porto Alegre, Rio Grande do Sul, Brasil, 2021

Structure and presentation	Extremely important	Very important	CVI
Language suitable for the target audience	2	6	1.0
Appropriate language for educational material	2	6	1.0
interactive language	3	5	1.0
correct information	1	7	1.0
objective information	1	7	1.0
enlightening information	2	6	1.0
Necessary informations	2	6	1.0
Logical sequence of ideas	0	8	1.0
current topic	1	7	1.0
Appropriate text size	1	7	1.0

*CVI= Content Validity Index.*

Regarding the relevance of the booklet, 16 (66.6%) responses were marked as extremely relevant, according to data in Table 3.

**Table 3:** Assessment of the judges regarding the relevance of the educational material (N=08). Porto Alegre, Rio Grande do Sul, Brazil, 2021

Importance	Extremely important	Very important	CVI
Stimulates learning	2	6	1.0
Contributes to knowledge in the area	3	5	1.0
Stimulates interest in the topic	3	5	1.0

*CVI= Content Validity Index.*

With regard to suggestions and improvements to the booklet, only two judges brought considerations, namely: including specific guidance on the myocardial revascularization procedure such as the vessels used for the “bridge” and images of the types of valve prosthesis, making adjustments in writing and include care related to dental procedures. All suggestions have been carried out.

Validation by the target audience took place with ten hospitalized patients, with a mean age of 53.8±6.14 years, 80% of whom were male. Of the total of 10 patients, 5 underwent CABG and 5 valve surgeries. Table 4 presents the results regarding the organization of the material, writing style, appearance and motivation.

**Table 4:** Evaluation of the target audience regarding the organization, writing style, appearance and motivation of the booklet (N=10). Porto Alegre, Rio Grande do Sul, Brazil, 2021

Items	Positive response	Negative response	AI
Organization			
Did the cover catch your attention?	10	0	1.0
Is the sequence of the content adequate?	10	0	1.0
Is the structure of the educational booklet organized?	10	0	1.0
Writing style			
As for the understanding of the sentences, they are: (Easy to understand/Difficult/Don't know)	10	0	1.0
Written content is:	10	0	1.0
(Clear/Confused/Don't know)	10	0	1.0
The text is: (Interesting/Uninteresting/Don't know)	10	0	1.0
Writing style and appearance			
The illustrations are: (Interesting/Uninteresting/Don't know)	8	2	0.8
Do the illustrations serve to complement the text? (Yes/No/Don't know)	10	0	1.0
Do the pages or sections look organized? (Yes/No/Don't know)	10	0	1.0
Motivation			
In your opinion, will anyone in the postoperative period of heart surgery who reads this booklet understand what it is about? (Yes/No/Don't know)	10	0	1.0
Did you feel motivated to read the booklet until the end? (Yes/No/Don't know)	10	0	1.0
Does the educational material address the issues necessary for people in the postoperative period of heart surgery to provide adequate care? (Yes/No/Don't know)	10	0	1.0
Did the educational booklet suggest you to act or think about self-care after cardiac surgery? (Yes/No/Don't know)	10	0	1.0

*AI: agreement index.*

There were two negative responses in the target audience’s evaluations, obtaining AI 0.8 in the illustrations category. In the same item, one patient answered that he does not know and another as uninteresting.

## DISCUSSION

This study presents the construction and validation of an educational booklet for patients in the postoperative period of cardiac surgery, used as a support resource for guidance and preparation for hospital discharge. The construction of this educational tool was based on specialized literature and validated with health professionals and patients themselves. Receiving guidance about postoperative care, with the support of educational materials, converges with the objectives of a satisfactory recovery that aims at the absence of complications and gradual return to daily activities^(22)^. Educational technologies are necessary and significant, as they have the objective of improving knowledge, stimulating autonomy, contributing to self-care, inserting the individual in the teaching and learning processes^(23)^. In the hospital environment, where most of the educational actions are still verbal, that is, professionals who guide patients and families, it becomes necessary to use other resources in order to contribute to the different degrees of retention induced by various types of learning^(13)^. Educational actions should be individualized and person-centered in order to ensure that patients’ educational needs are met. This empowers patients by increasing self-efficacy or confidence, resulting in autonomy and a smoother discharge process^(24)^.

A study carried out with 50 patients undergoing cardiac surgery showed that among the topics related to the postoperative period, to which patients do not know how to respond, are food and diet, return to daily activities, practice of physical activities, sexual activity, care for the surgical wound and identification of signs of infection^(13)^. Another study carried out with 90 patients undergoing CABG showed that the most common doubts are related to the surgical wound of the chest and leg, complications arising from the procedures, medications, anatomical and physiological factors and information about medication and diet^(14)^. In addition to these topics, an integrative review that evaluated six studies related to the topic included psychological symptoms, control of risk factors and clinical signs in its result synthesis^(25)^.

The booklet developed in this study addresses the items mentioned, except for medications in general, since this varies according to each patient. However, the booklet addresses oral anticoagulation, which is usually indicated for patients undergoing valve surgery and requires special care. Preparing for hospital discharge should also include guidance on emotional aspects, which may lead to symptoms of sadness and depression, fatigue, anxiety, feelings of vulnerability and, consequently, changes in sleep patterns^(12)^. Thus, it is important to include patients’ relatives so that they can support them in these moments. And resources such as the printed booklet, which includes the relevant guidelines for home care, can be useful to the family.

Given the above, during the preparation of support materials aimed at health education, it is important to submit them to validation by expert judges who evaluate each of the items to determine whether they represent the domain of interest. Added to this is validation by the target audience, along with material that is in line with the demands and needs of patients and families. With the validation process, the quality of the educational material for the target audience is sought, prioritizing the quality and clarity of the information. Thus, when participating in the study, the judges agreed with the applicability of the educational material for the practice of safe discharge. In this study, the judges evaluated the booklet in relation to its objectives, structure/presentation and its relevance, and all items were evaluated as relevant. The suggestions referred to the need to add some differences between the types of “bridges” performed in CABG (mammary and saphenous), the types of prostheses used in valve surgery (biological and metallic) and care when undergoing dental procedures, as in addition to the need for prior suspension of the anticoagulant in use, there is a risk of infection with complications that may involve the heart valves. Such suggestions were added in the texts of the item’s myocardial revascularization surgery, valve surgery and anticoagulation.

As for the validation of the target audience, a satisfactory evaluation was obtained regarding writing, appearance and motivation. Only one patient analyzed the item illustrations as uninteresting and the other impartially. Even so, most patients considered the use of the booklet to promote health education, focused on post-discharge care, valid. It is important to mention that two figures were modified, considering the profile of surgical patients. Validation of educational materials

### Study limitations

The selection of articles and the interpretation of information for the construction of the booklet may be subject to the subjective interpretation and context of the authors’ work.

### Contributions to the Nursing area

This study brings together the main topics that should be included in the guidelines for the discharge of patients undergoing cardiac surgery, both myocardial revascularization and valve surgery, validated by professionals experienced in the subject and by the patients themselves.

## CONCLUSION

The educational booklet for patients undergoing cardiac surgery was built with 14 topics related to the care needed for recovery after hospital discharge and validated with experienced professionals and by the patients themselves. The final printed version, called Post-Cardiac Surgery Guidelines, is used as a support resource for the guidelines provided by nurses and physicians during preparation for hospital discharge, serving as reference material for patients and family members at home.
